# Myo-inositol therapy for poor-responders during IVF: a prospective controlled observational trial

**DOI:** 10.1186/s13048-015-0167-x

**Published:** 2015-06-12

**Authors:** Francesca Caprio, Maria Diletta D’Eufemia, Carlo Trotta, Maria Rosaria Campitiello, Raffaele Ianniello, Daniela Mele, Nicola Colacurci

**Affiliations:** Outpatient Fertility Clinic, Second University of Naples, Largo Madonna delle Grazie 1, Naples, 80138 Italy

**Keywords:** Myo-inositol, Poor-responder, IVF, Ovarian sensitivity index

## Abstract

**Background:**

The overall incidence of poor ovarian response in IVF cycles has been reported to be between 9 and 24 %. The management of these patients remains a significant challenge in assisted reproduction. The aim of the present study was to evaluate the effect of myo-inositol (MI) on ovarian function in poor responders undergoing ICSI.

**Methods:**

The study is a prospective controlled observational trial, that involved 72 poor responders included in an ICSI program and divided into two groups; group A: 38 patients who have been assuming MI (4 g) + folic acid (FA) (400 μg) for the previous 3 months before the enrollment day; group B: 38 patients assuming FA (400 μg) alone for the same period. COH was carried out in the same manner in the two groups. The main goal was the assessment of oocytes retrieved number and quality; secondary endpoints were the Ovarian Sensitivity Index (OSI: n° oocytes retrieved/total Gonadotropins units × 1000), oestradiol levels on the day of hGC, fertilization rate, implantation rate, ongoing pregnancy rate.

**Results:**

There was no significant difference between the two groups regarding oestradiol level, but total rec-FSH units were significantly lower (*p* = 0.004) and M2 oocytes rate significantly higer (*p* = 0.01) in group A. The ovarian sensitivity index was higher, reaching a statistical significance (*p* < 0.05), in the group of patients pre-treated with MI, showing an improvement in ovarian sensibility to gonadotropin.

**Conclusions:**

Our results suggest that MI therapy in poor responders results in an increased of the number of oocytes recovered in MII and of the gonadotropin Ovarian Sensitivity Index (OSI), suggesting a MI role in improving ovarian response to gonadotropins. Therefore MI seems to be helpful in “poor responders” undergoing IVF cycles.

## Background

It is well known that advanced reproductive age patients exhibit worst response to ovulation induction because female age is the main factor that affects the number of retrieved oocyte and gonadotropins set [1].

In addition to a quantitative reduction of their oocyte cohorts, poor ovarian responder patients (PORs) present a high risk of implantation failure that may suggest a compromised oocyte quality [2].

The overall incidence of poor ovarian response has been reported to be between 9 and 24 % [3, 4]. A variety of regimens have been employed, including the use of increased gonadotropin doses, low dose gonadotropin-releasing hormone (GnRH) agonists or antagonists, flare regimes, sequential protocols, adjunctive growth hormone, dehydroepiandrosterone adjuvant therapy, minimal ovarian stimulation with clomiphene citrate, and natural cycle IVF [5–8]. However, the ideal stimulation regimen for poor responders is currently unknown. In addition, there is insufficient evidence to support the use of specific interventions to improve IVF treatment outcomes in poor responders. The Cochrane review of poor responder interventions concluded that no particular treatment offered clear benefit, or could be recommended [4]. The management of poor responders therefore remains a significant challenge in assisted reproduction.

Inositol is a sugar-similar molecule, which has been erroneously included among vitamins for a long time, while it is a vitamin-factor of B group.

Many studies support the notion that myo-inositol (MI), an inositol isoform, is one of the precursors for the synthesis of phosphatidylinositol polyphosphates (PIPs). PIPs are key biomolecules considered part belonging to the signal transduction system known to be involved in the regulation of several cellular functions [9]. Infact, MI plays a crucial role in signal transduction, cell morphogenesis and cytogenesis, it is involved in cell membrane formation, lipid synthesis and cell growth [10].

The role of inositol in the streamlining of ovulatory process has been widely defined in patients with reduced sensitivity to insulin, as far as the inositol’s insulin sensitizing action [11]. Recently it has been hypothesized that inositol could have a different way of action on different cell kinds, apart from the insuline-resistance reduction mechanism. Indeed, at ovarian level MI has been shown to be crucial for the FSH signaling and it is involved in oocyte maturation and embryo development. Recently, an international consensus conference, further confirmed that, during IVF program, MI pretreatment is able to improve the oocyte and embryo quality [12, 13].

A previous study has demonstrated that supplementation with inositol can reduce the oxidative stress caused by different agents through the induction of natural antioxidant defenses by increasing superoxide dismutase (sod) and catalase (cat) levels and intracellular content of glutathione (GSH) [14].

Noteworthy, the data discussed in the consensus manuscript highlighted that MI pre-treatment is effective on both PCOS and healthy women undergoing controlled ovarian hyperstimulation.

On this basis, inositol is becoming part of clinical practice in the treatment of both PCO and not PCO women, both for its insulin-sensitizing activity and its effect on oocyte maturation and quality [15].

Nevertheless, data are missing on the use of MI in women defined poor responders undergoing ovarian stimulation for ICSI cycle. Therefore, the aim of the present study was to provide evidence on the matter and eventually open new research line.

## Materials and methods

### Patients

The study is a prospective controlled observational trial, that involved 76 women included in an ICSI program at the IVF Unit of the Second University of Naples between November 2013 and November 2014. All the women were poor-responder, according to the Bologna Criteria, defined by the Consensus Group for the European Society for Human Reproduction and Embryology (ESHRE) to help assigning more uniform patient groups in clinical trials [16]. Poor responders were classified with at least two of the three following criteria: (I) advanced maternal age (>40 years) or any other risk factor for POR; (II) a previous POR (<3 oocytes with a conventional ovarian stimulation protocol); and (III) an abnormal ovarian reserve test (ORT).

According to our experience we have considered the ovarian reserve test altered in case of AFC <5–7 follicles or AMH <1.5 ng/ml.

The selection criteria for this study were as follows: women of 30 to 42 years old, with normal ovulatory cycles of 24 to 35 days in length, FSH value <15 IU/mL, body mass index (BMI) between 18 and 25 kg/m^2^ and absence of any endocrine and metabolic disorders such as polycystic ovary syndrome, hyperprolactinemia, diabetes and thyroid dysfunction.

Patients were excluded from this study if they were found to have any significant pelvic pathology such as hydrosalpinx, uterine anomaly, advanced endometriosis of stage III to IV or fibroids with uterine cavity distortion. Patients with a partner with a severe oligo-astheno-teratozoospermia (OAT) or azoospermia were excluded, as well.

Patients satisfying all the inclusion criteria were included in the study and divided into two groups: group A consisted of 38 patients who have been assuming 4 g of MI + 400 μg of folic acid (Inofert, Italfarmaco Spa, Milan) every day for the previous 3 months before the enrollment day; group B consisted of 38 patients assuming 400 μg of folic acid (Folidex, Italfarmaco Spa, Milan) alone every day for the same period. The MI dosage was evaluated according to a previous study that investigated the effect of a supplementation with MI and melatonin on oocyte quality in women who failed to conceive in previous in vitro fertilization cycles for poor oocyte quality [17].

### Stimulation protocol

Patients underwent controlled ovarian hyperstimulation (COH) with the use of a GnRH antagonist protocol. COH was carried out in an identical manner in the two groups, with 300 IU of recombinant FSH (Gonal-F, Merck-Serono, Switzerland), from day 2 or 3 of the cycle. Daily injections of a GnRH antagonist (Cetrotide 0.25 mg sc, Merck-Serono, Switzerland) were administered to prevent premature ovulation by using the flexible antagonist protocol, according to a personalized regimen, from the day the leading follicle reached 14 mm in diameter until the day of hCG injection. Ovulation induction was monitored by vaginal ultrasound and hormonal assessment every second–third day. When at least two follicles had reached a diameter of 18 mm, a single i.m. bolus of 10.000 IU of hCG (Gonasi HP 5000; IBSA, Rome, Italy) was administered. Transvaginal follicular aspiration was performed 34–36 h after hCG administration.

The experimental study was conducted in accordance with principles of the Helsinki Declaration of 1975, using routine clinical practice procedures usually performed during IVF cycles; such procedures did not involve additional risks to the patients and all the medical decisions concerning individual patients were not affected by the study. The Institutional Review Board approved the protocol and all patients gave a written informed consent before entering in the study.

### Laboratory procedures

Oocytes were cultured in Petri dishes in IVF-20 (Vitrolife, Göteborg, Sweden) at 37 °C in a humidified 5 % carbon dioxide/95 % air environment. The semen was processed with 80 % Percoll (Sigma Chemical Company, St. Louis, MO) discontinuous gradient centrifugation at 800 g for 15 min. ICSI procedures have been described in detail previously [18, 19] and were performed 4–6 h after oocyte retrieval. After IVF, the resulting embryos were cultured in IVF-20 at 37° under 5 % carbon dioxide in air. Embryos were graded by morphological analysis: grade 1, blastomeres with 5–10 % extracellular fragmentation; grade 2, 10–20 % extracellular fragmentation; grade 3, 20–30 % extracellular fragmentation; grade 4, >30 % blastomeric fragmentation.

The luteal phase was supported with the administration of 33 mg/day of natural progesterone starting from pick-up day and then 50 mg/day from embryo transfer day. Serum levels of hCG were measured 14 days after embryo transfer and, if positive, were obtained every 3–6 days until an intrauterine gestational sac was demonstrated by US examination.

The main goal of the study was the definition of the number and the quality of the oocytes retrieved; in particular the rates of M2 oocytes and unsuitable oocytes (only zona, M1 oocytes, germinal vescicle and degenerated oocytes) were evaluated.

Secondary endpoints were the Ovarian Sensitivity Index (OSI: n° oocytes retrieved/total Gonadotropin units × 1000), oestradiol levels at the day of hGC administration, fertilization rate, implantation rate, ongoing pregnancy rate.

## Statistical analysis

Data were analysed using the Statistical Package for the Social Science (SPSS Release 16.0.2; SPSS Inc., Chicago, IL). Continous variables were presented as mean and SD. Statistical analysis was performed using an unpaired *t*-test for independent data and *t*-test, setting the significance level at *p* ≤ 0.05.

## Results

Group A and B were homogeneous for age and causes of infertility of their members. Baseline characteristics of patients were similar (Table 1). There was no significant difference between the two groups regarding oestradiol level, but total rec-FSH units were significantly lower in group A (1975 ± 298 vs 2212 ± 312, *p* = 0.004) (Table 2). The mean number of oocytes retrieved was similar in the two study groups, but the results show a statistically significant increase of M2 oocytes rate in group A (80.5 % vs 66.6 %, *p* = 0.01). The rate of oocytes that did not show the suitable features for insemination (only zona, M1 oocytes, germinal vescicle and degenerated oocytes) was statistically significantly lower in group A (19.4 % vs 33.3 % *p* = 0.01) (Table 3). The fertilization rate (87 % vs 84 %), implantation rate (10.8 % vs 9 %), grade 1 embryos rate (87.9 % vs 81.9 %) and pregnancy rate (18.4 % vs 15.7 %) showed a positive trend in patients pretreated with MI, without reaching a statistical significance (Table 3).

**Table 1:** Baseline characteristics

	Group A	Group B	*P*
	(35 patients)	(30 patients)	
Age (years)	33.2 ± 2.8	33.9 ± 3.1	NS
Body mass index (kg/m^2^)	23.3 ± 2.3	24.2 ± 2.0	NS
Time of infertility (years)	1.9 ± 1	2.2 ± 0.5	NS
Day-3 FSH (IU/mL)	7.4 ± 1.9	6.9 ± 2.3	NS
AMH (ng/mL)	2.3 ± 1.1	2.8 ± 1.8	NS

Data are presented as mean ± SD (range). *P* not significant (NS) for all comparisons (*P* > 0.05)

**Table 2:** Stimulation data

	Group A	Group B	*p*
Oestradiol level	1129 ± 366	1016 ± 462	n.s
rec-FSH (U.I./mL)	1975 ± 298	2212 ± 312	<0.05
n° oocytes retrieved	3.65 ± 1.32	3.39 ± 1.38	n.s
O.S.I.	1.88 ± 0.81	1.54 ± 0.65	<0.05

*n.s.* not significant, *O.S.I.* ovarian sensitivity index

**Table 3:** Oocytes quality, embryo characteristics and clinical outcome

	Group A	Group B	*p*
No. of oocytes M2 (%)	80,5 %	66.6 %	<0.05
No. of not suitable oocytes^a^ (%)	19,4 %	33.3 %	<0.05
Fertilization rate (%)	87 %	84 %	n.s
Implantation rate (%)	10,8 %	9 %	n.s.
Grade I embryo rate (%)	87.9 %	81,9 %	n.s
Pregnancy rate (%)	18,4 %	15.7 %	n.s.

^a^M1, Germinal Vescicle, only zona and degenerated oocytes

The ovarian sensitivity index was higher, reaching a statistical significance, in the group of patients pre-treated with MI plus folic acid than in the control group (1.88 ± 0.81 vs 1.54 ± 0.65, *p* < 0.05), showing an improvement in ovarian sensibility to gonadotropin (Table2).

## Discussion

This pilot study evaluates the impact that a treatment with MI could have on patients commonly defined poor responders on the outcome of IVF cycles.

The fertilization rate, implantation rate, grade 1 embryos rate and pregnancy rate showed a positive trend in patients pretreated with MI, without reaching a statistical significance.

The results suggest that MI therapy is associated to an increase in M2 oocytes retrieved number and in ovarian sensitivity to gonadotropins in poor responders, in which IVF is burdened by high dosages of gonadotropins and the low oocyte number and quality affects and limits the results of the technique.

The first study about the role of MI in in-vitro human fertilization (IVF) dates back to 1992. Such study reported an elevated level of inositol in serum samples of patients having successful IVF pregnancies, thus indicating a possible involvement of inositol in both the early in vitro phase of IVF and the maintenance of normal embryonic development [20]. A later study demonstrated that a higher concentration of MI in human follicular fluid positively correlates with good oocytes quality [21].

Furthermore, it was demonstrated that supplementation with MI is positively related to meiotic progression of mouse germinal vesicle oocytes, through the enhancement of intracellular calcium oscillation [22].

Previous experiences had already shown the beneficial effects of MI-based treatment on oocyte quality in PCO women, suggesting a possible beneficial effect of MI on oocyte competence and maturation [13, 23].

The beneficial effect of the co-treatment with inositol and melatonin in managing patients with a low oocyte quality in IVF cycles [17] has been also shown, even if no potential positive effect–directly related to inositol–for poor responding patients has been proven so far.

However, this pilot study has some limitations: the number of patients involved is too small to offer a great significance of the result obtained and we have not analyzed other potential factors that may have affected the differences in ovarian sensitivity (as polymorphisms of FSH receptor).

## Conclusion

In conclusion, our results, related to other study reported above, suggest that MI could plays a role in oocyte maturation and competence, and its concentration could even influence cellular homeostasis.

Although MI supplementation had not a statistical significance effect on pregnancy rate, the group pre-treated with MI showed a significant increase of the gonadotropin ovarian sensitivity index (OSI), resulting from the ratio between the number of oocytes obtained and the unities of gonadotropin used. This latter result suggests a new role for MI in the intracellular signal-transduction pathways mediated by the gonadotropin receptor, supporting the development of new research perspectives and new therapeutic strategies for the management of poor-responders patients. Additional, larger randomized controlled studies are needed to reinforce our preliminary findings.
